# Uncovering anthocyanin biosynthesis related microRNAs and their target genes by small RNA and degradome sequencing in tuberous roots of sweetpotato

**DOI:** 10.1186/s12870-019-1790-2

**Published:** 2019-06-03

**Authors:** Liheng He, Ruimin Tang, Xiaowen Shi, Wenbing Wang, Qinghe Cao, Xiayu Liu, Ting Wang, Yan Sun, Hongmei Zhang, Runzhi Li, Xiaoyun Jia

**Affiliations:** 1 Shanxi Agriculture University, Taigu, 030801 Shanxi China; 2 Jiangsu Xuzhou Sweetpotato Research Center, Xuzhou, 221131 Jiangsu China; 3 Maize Research Institute, Shanxi Academy of Agricultural Sciences, Xinzhou, China

**Keywords:** Anthocyanin biosynthesis, Degradome analysis, MiR156, Small RNA sequencing, Sweetpotato

## Abstract

**Background:**

Compared with white-fleshed sweetpotato (WFSP), purple-fleshed sweetpotato (PFSP) is a desirable resource for functional food development because of the abundant anthocyanin accumulation in its tuberous roots. Some studies have shown that the expression regulation mediated by miRNA plays an important role in anthocyanin biosynthesis in plants. However, few miRNAs and their corresponding functions related to anthocyanin biosynthesis in tuberous roots of sweetpotato have been known.

**Results:**

In this study, small RNA (sRNA) and degradome libraries from the tuberous roots of WFSP (Xushu-18) and PFSP (Xuzishu-3) were constructed, respectively. Totally, 191 known and 33 novel miRNAs were identified by sRNA sequencing, and 180 target genes cleaved by 115 known ib-miRNAs and 5 novel ib-miRNAs were identified by degradome sequencing. Of these, 121 miRNAs were differently expressed between Xushu-18 and Xuzishu-3. Integrated analysis of sRNA, degradome sequencing, GO, KEGG and qRT-PCR revealed that 26 differentially expressed miRNAs and 36 corresponding targets were potentially involved in the anthocyanin biosynthesis. Of which, an inverse correlation between the expression of ib-miR156 and its target *ibSPL* in WFSP and PFSP was revealed by both qRT-PCR and sRNA sequencing. Subsequently, ib-miR156 was over-expressed in *Arabidopsis*. Interestingly, the ib-miR156 over-expressing plants showed suppressed abundance of *SPL* and a purplish phenotype. Concomitantly, upregulated expression of four anthocyanin pathway genes was detected in transgenic *Arabidopsis* plants. Finally, a putative ib-miRNA-target model involved in anthocyanin biosynthesis in sweetpotato was proposed.

**Conclusions:**

The results represented a comprehensive expression profiling of miRNAs related to anthocyanin accumulation in sweetpotato and provided important clues for understanding the regulatory network of anthocyanin biosynthesis mediated by miRNA in tuberous crops.

**Electronic supplementary material:**

The online version of this article (10.1186/s12870-019-1790-2) contains supplementary material, which is available to authorized users.

## Background

Sweetpotato (*Ipomoea batatas* L.), a hexaploid (2n = 6x = 90) dicotyledonous plant of *Convolvulaceae* family, is an important crop around the world due to its high yield, wide adaptability and rich nutrition [1]. The flesh of the tuberous roots has multiple colors, such as white, yellow, orange and purple [2]. The purple-fleshed sweetpotato (PFSP) is not only as nutritious as the white-fleshed sweetpotato (WFSP) but also enriches high content of anthocyanin. Anthocyanin is water-soluble pigment belonging to the flavonoid group. Numerous studies have showed that anthocyanin has a strong antioxidant activity and therefore has therapeutic effects on a variety of diseases like obesity and cancers [3]. More importantly, the anthocyanin of PFSP has high thermal stability and light stability with a high level of acylation than that of strawberry, raspberry, and apple [4–6]. Therefore, the PFSP has been recently proposed as a potential pharmaceutical crop for developing drugs, such as antineoplastic, antiinflammatory, and antioxidant agents [6, 7].

Anthocyanin biosynthetic pathway has been extensively studied in plants [8, 9]. Genes that participate in anthocyanin biosynthesis can be classified into structural genes and regulatory genes. The structural genes encode a series of enzymes, including phenylalanine ammonialyase (*PAL*), chalcone synthase (*CHS*), chalcone isomerase (*CHI*), flavonoid 3′-hydroxylase (*F3’H*), flavonoid-3′5’-hydroxylase (*F3’5’H*), flavanone 3-hydroxylase (*F3H*), dihydroflavonol reductase (*DFR*), UDP-glucose flavonoid 3-o-glycosyltransferase (*UFGT*), and anthocyanidin synthase (*ANS*) [10]. These enzyme genes could be transcriptionally regulated by MYB-bHLH-WDR (MBW) complexes consisting of three kinds of regulatory factors, MYB, bHLH and WD40 repeat [11]. Additionally, other regulatory genes have also been reported to regulate anthocyanin biosynthesis, such as Constitutively photomorphogenic1 (*COP1*) [12], Jasmonate zim-domain (*JAZ*) [13], the Squamosa promoter binding proteinlike (*SPL*) [14], and *NAC* [15]. Apart from the structural genes and regulatory genes, recent evidences showed that microRNAs (miRNAs) can also play important roles in mediating anthocyanin biosynthesis in plants [16–18].

MiRNA, widely existed in eukaryotes, is a kind of endogenous non-coding small RNA with approximately 20–24 nt in length. Numerous evidences showed that miRNAs participated in regulating gene expression mainly via cleaving target mRNAs or preventing gene translation at the post-transcriptional level [19, 20]. In plants, miRNAs are involved in multiple biological processes, including growth and development [12], stress responses [21, 22], auxin signaling [23, 24] as well as secondary metabolism. For example, miR156, miR165/166, miR828 and miR858, have been identified to be involved in anthocyanin biosynthesis in *Arabidopsis* [14, 16, 17, 25, 26] and *Solanum lycopersicum* [18]. By high throughput sequencing, more and more miRNAs have been identified and characterized in wheat [27, 28], soybean [29], rice [30], sweet orange [31], asparagus [32], chinese radish [33], maize [34], tea [35] etc. So far, a total of 38,589 miRNAs from 271 different plants were recorded in the latest miRBase database (http://www.mirbase.org/). A couple of sweetpotato miRNAs and their targets have also been identified [36, 37]. However, miRNAs involved in regulating anthocyanin biosynthesis in sweetpotato have not been systematically reported.

In this study, sRNA and degradome sequencing were used to identify miRNAs and their corresponding targets that potentially involved in anthocyanin biosynthesis in sweetpotato. Small RNA and degradome libraries from the tuberous roots of WFSP (cultivar Xushu-18, XS-18) and PFSP (cultivar Xuzishu-3, XZS-3) were constructed and sequenced. Integrated analysis of sRNA, degradome sequencing, GO, KEGG and qRT-PCR revealed a comprehensive account of the ib-miRNA populations, corresponding targets, expression abundance, as well as miRNAs potentially involved in the anthocyanin metabolism. Expression levels of eight differently expressed miRNAs and their targets were validated by qRT-PCR, which were consistent with the sequencing data. In addition, we demonstrated that the over-expression of ib-miR156 in *Arabidopsis* strongly suppressed the abundance of *ibSPL* and resulted in a purplish phenotype. Finally, a possible ib-miRNA-target regulatory model associated with anthocyanin biosynthesis in tuberous roots of sweetpotato was illustrated. Our findings provided a comprehensive expression profiling of ib-miRNAs, and suggested that miRNAs were involved the regulation of anthocyanin biosynthesis in sweet potato.

## Result

### Small RNA populations in the tuberous roots of sweetpotato

**Fig. 1:**
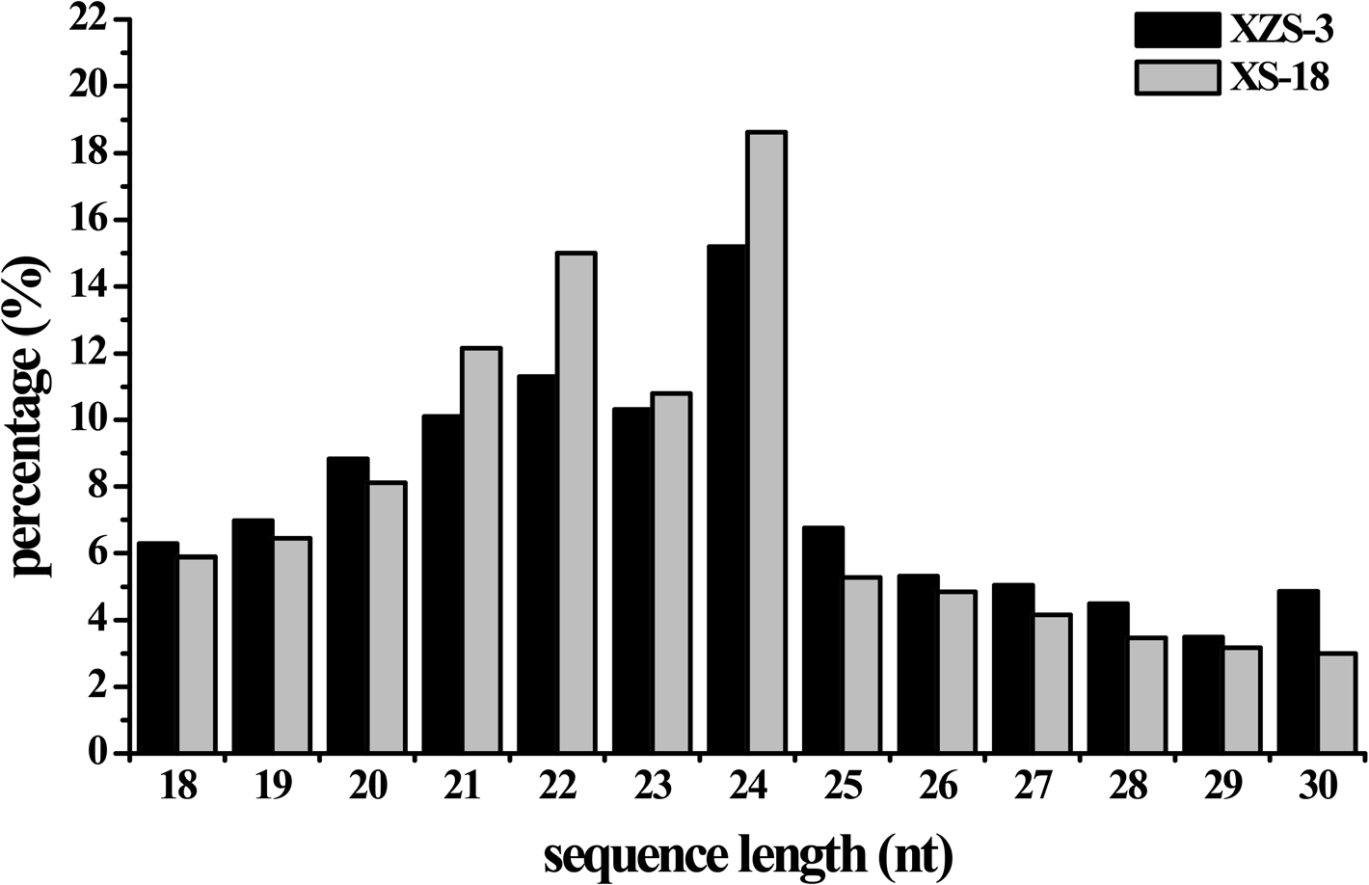
Length distribution of sRNAs in XS-18 and XZS-3 libraries

To identify miRNAs involved in the process of anthocyanin biosynthesis, the tuberous roots of WFSP (XS-18) and PFSP (XZS-3) were used to construct sRNA libraries and sequenced on Illumina HiSeq2000 platform, respectively. A sum of 27,705,914 (XS-18) and 28,947,914 (XZS-3) raw reads were produced. After removing of the low quality reads and contaminated adapter sequences, 26,825,634 (96.82%) and 27,331,707 (94.42%) clean reads were obtained from XS-18 and XZS-3, respectively. A total of 15,764,489 (XZS-3, 91.61%) and 13,904,486 (XS-18, 73.32%) sRNAs were mapped to the corresponding assembled unigenes of sweetpotato (Additional file 1). The mapped reads were further annotated against Pfam database, and subsequently divided into rRNAs, tRNAs, snRNAs, snoRNAs, ta-siRNA (TAS) and others. The endogenous sRNAs were identified as known miRNAs and novel miRNAs (Additional file 2). The length distribution patterns of the sRNAs were similar in the two libraries. They were ranged from 18 to 30 nt, of which 24 nt sRNAs were the most abundant size with 18.63 and 15.19% in XS-18 and XZS-3 libraries, respectively (Fig. 1).

### Identification of known and novel ib-miRNAs in sweetpotato

**Fig. 2:**
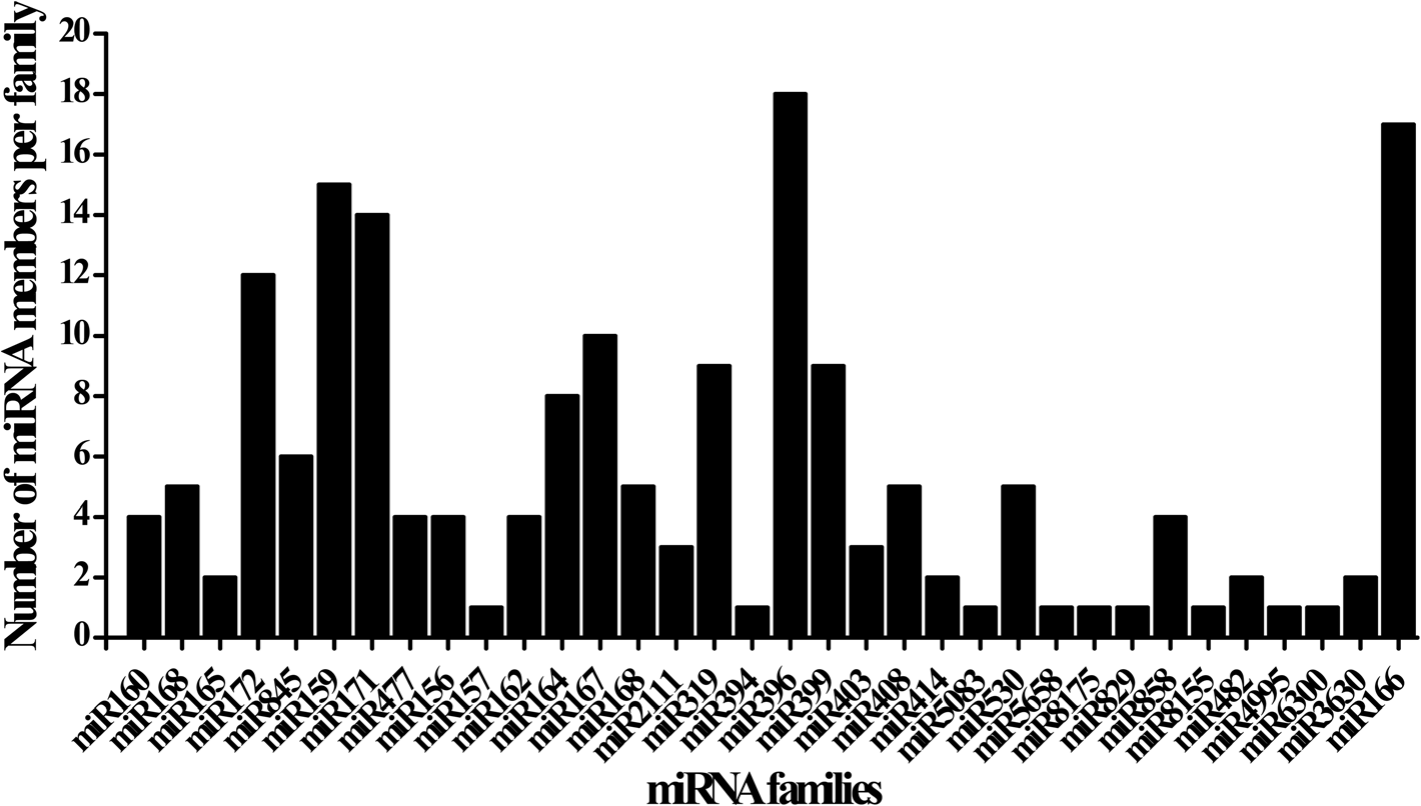
Member numbers of known miRNA families identified in sweetpotato

The known miRNAs were found to be important in plant growth, development and many other biological processes [38]. To identify known miRNAs in sweetpotato, the sRNA sequences were aligned to the mature miRNA sequences deposited in the miRBase 22.1. A total of 191 known miRNAs belonging to 43 miRNA families were identified in the two libraries, with 185 and 145 known miRNAs in XS-18 and XZS-3, respectively (Additional file 3). Among the 43 identified families, the member number of each miRNA family varied significantly. The ib-miR396 family contained the largest number with 18 members, followed by ib-miR166 and ib-miR159 families, with 17 and 15 members, respectively. By contrast, 9 families had only one member, such as ib-miR157, ib-miR394, ib-miR5083 and ib-miR5658 (Fig. 2).

The abundance of the miRNAs were estimated as transcripts per million (TPM), which highly varied among the 43 known miRNA families (Additional file 4). Some miRNAs highly expressed with more than 1000 TPM, such as ib-miR159a-1 and ib-miR319a-1, whereas some miRNAs expressed at a lower level with less than 2 TPM, such as ib-miR160–1, ib-miR1030a-j and ib-miR1128. In addition, different members in the same miRNA family exhibited different expression levels. For instance, in miR159 family, ib-miR159a-1 showed the highest abundance with 5978 and 1106 TPM in the XS-18 and XZS-3 libraries, respectively. However, ib-miR159f only presented 4 and 1 TPM in the XS-18 and XZS-3 libraries, respectively (Additional file 4).

**Fig. 3:**
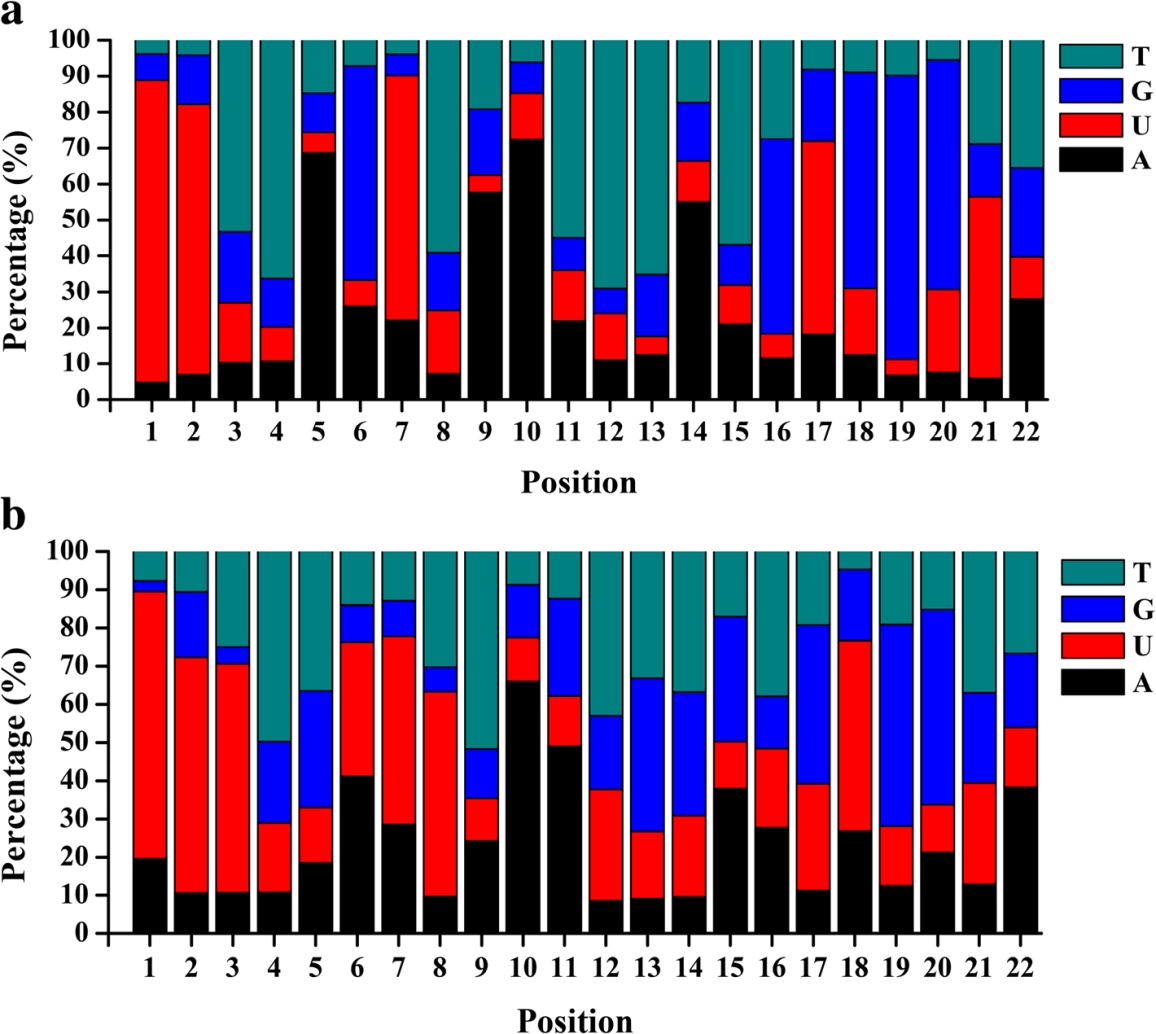
Relative nucleotide bias for each position of known **a** and novel miRNAs **b**

The length of known miRNAs ranged from 18 nt to 24 nt, in which 21 nt miRNAs were the most abundant size (Additional file 3). To understand the base preference of sweetpotato miRNAs, we analyzed the base distribution for each position of known miRNAs. In both libraries, nucleotide sequences analysis revealed that uridine (U) was the most common nucleotide at the 5′ end. The cleavage site of the target gene usually matched with the 10 th or 11 th nucleotide of miRNA. The majority nucleotides of known ib-miRNAs was adenine (A) at position 10 th and thymine (T) or A at position 11 th (Fig. 3a).

The remaining sRNA sequences were mapped to sweetpotato transcriptome database, and the hairpin structures were used to identify novel miRNAs in sweetpotato. Novel miRNAs were predicted based on the published criteria [39]. Totally, 33 and 33 novel miRNAs were predicted in the XS-18 and XZS-3 libraries, respectively (Additional file 5). The length distribution of the novel miRNAs was between 20 nt to 25 nt. Similar to known miRNAs, the nucleotide bias analysis showed that U appeared mainly at the 5′ end of novel miRNAs (Fig. 3b). The length of the novel miRNA precursors ranged from 57 nt to 293 nt, with an average length of 140 nt (Additional file 5), which was in accordance with the commonly observed length of miRNA precursors in plants [40]. The predicted hairpin structures of the novel miRNA precursors and locations of mature miRNAs in the precursors were shown in Additional file 6. The negative folding free energies of the hairpin structures ranged from − 116.21 to − 22.20 kcal mol^− 1^ with an average of − 57.62 kcal mol^− 1^. The minimal folding free energy index (MFEI) ranged from 0.51 to 2.44 with an average of 1.31, with most MFEIs being > 0.8 (Additional file 5). Usually, the presence of complementary miRNA star (miR*) strands lays strong support for the authenticity of novel miRNA [39]. Similar to the known miRNAs, the abundance of the identified novel ib-miRNAs in sweetpotato varied considerably. MiR* strands were found to be present in 17 of the 33 novel ib-miRNAs and expressed at lower levels than their corresponding miR strands (Additional file 5), which agreed with the report that miR* strands were mostly degraded [40].

### Target validation of ib-miRNAs by degradome sequencing

To explore the function of sweetpotato miRNAs, computational program psRNATarget (http://plantgrn.noble.org/v1_psRNATarget/) was performed to predict their target genes. A total of 3278 transcripts were predicted to be targets of the 224 miRNAs (Additional file 7). Then, high-throughput degradome sequencing was performed to validate the miRNA targets. A total of 1,886,192 unique reads of 14.51 million raw reads were obtained, and 766,328 unique reads matched with the reference sequences assembled from sweetpotato transcriptome database (Additional file 8). Totally, 180 target genes cleaved by 115 known ib-miRNAs and 5 novel ib-miRNAs were identified by CleaveLand4 analysis (http://sites.psu.edu/axtell/software/cleaveland4/) (Additional file 9).

The cleaved-target transcripts were classified into five categories, named category 0, 1, 2, 3 and 4, according to the relative abundance of the tags at the target sites. The miRNAs and corresponding targets in the five categories were shown in Additional file 9. Among the 180 identified targets, 55, 8, 34, 1 and 23 targets were found in categories 0, 1, 2, 3 and 4, respectively.

**Fig. 4:**
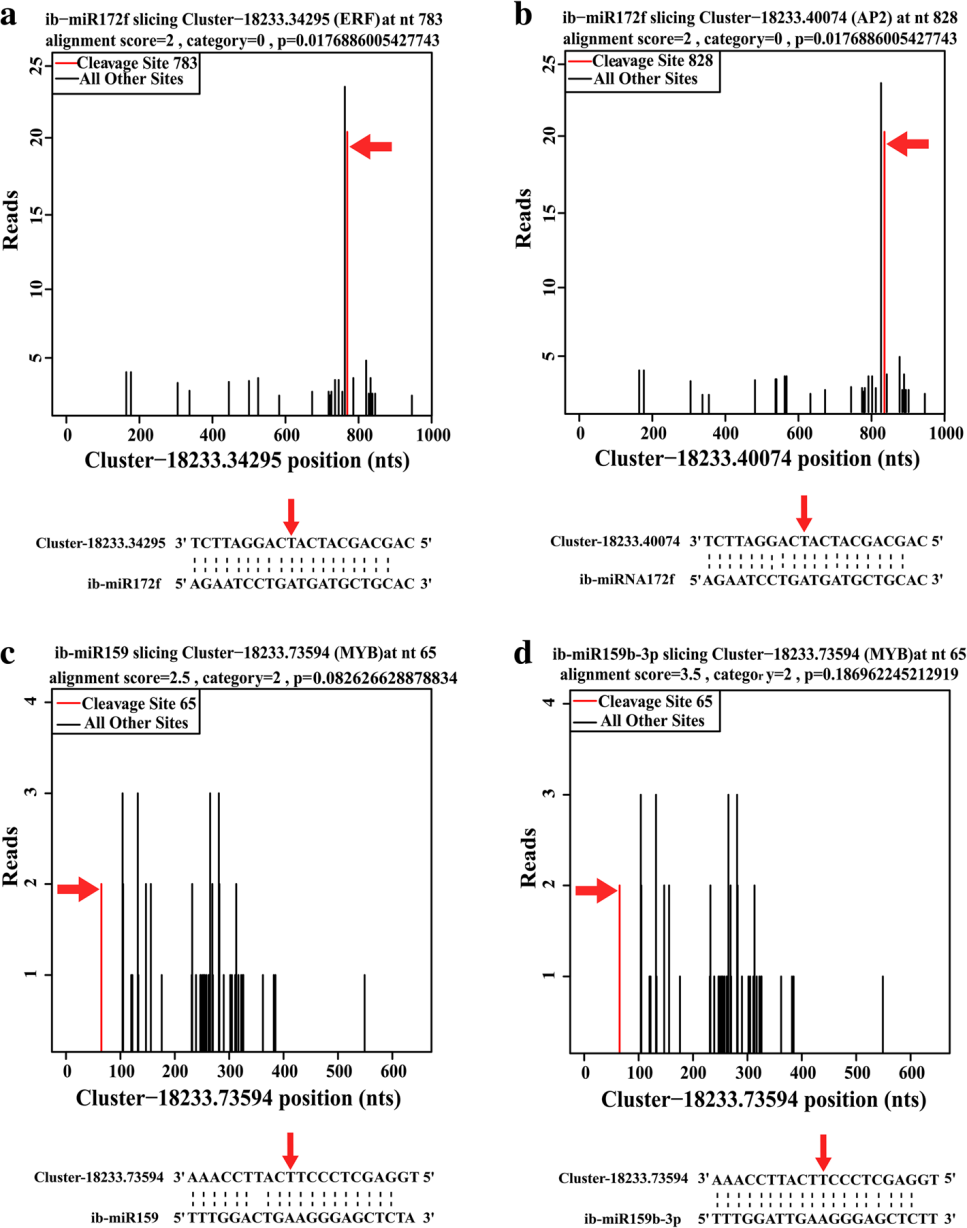
Target plot (t-plots) of representative ib-miRNA targets confirmed by degradome sequencing. The red lines showed the distribution of the degradome tags along the target mRNA sequences. The red arrows represented the cleavage nucleotide positions on the target genes. **a**, **b** Cleavage features in *ibERF* and *ibAPETALA2* mRNA by ib-miR172f. **c, d** Cleavage features in *ibMYB* mRNA by ib-miR159 and ib-miR159b-3p

Target analysis showed that many cleaved-target transcripts by ib-miRNAs were TF genes, including *MYB*, *WRKY*, *NAC*, *SPL*, *ARF*s, *ERF*, *WDR*, *HD-ZIPIII*, etc. (Additional file 9). Many ib-miRNAs had more than one transcript as target genes. For example, ib-miR172 could target *ERF* (*Cluster-18,233.34295, Cluster-18,233.74214*) and *APETALA 2* (*Cluster-18,233.40074, Cluster-18,233.74210*) (Fig. 4a, b). Furthermore, the same transcript was targeted by more than one ib-miRNA. For instance, members of ib-miRNA159 family, ib-miR159, ib-miR159a, ib-miR159b-3p and ib-miR159c shared the same target *ibMYB* (*Cluster-18,233.73594*) (Fig. 4c, d). *MYB* gene (*Cluster-18,233.73594*) was also targeted by ib-miR858, ib-miR319, ib-miR159 and ib-miR156 (Additional file 9).

### Differentially expressed ib-miRNAs between WFSP and PFSP

**Fig. 5:**
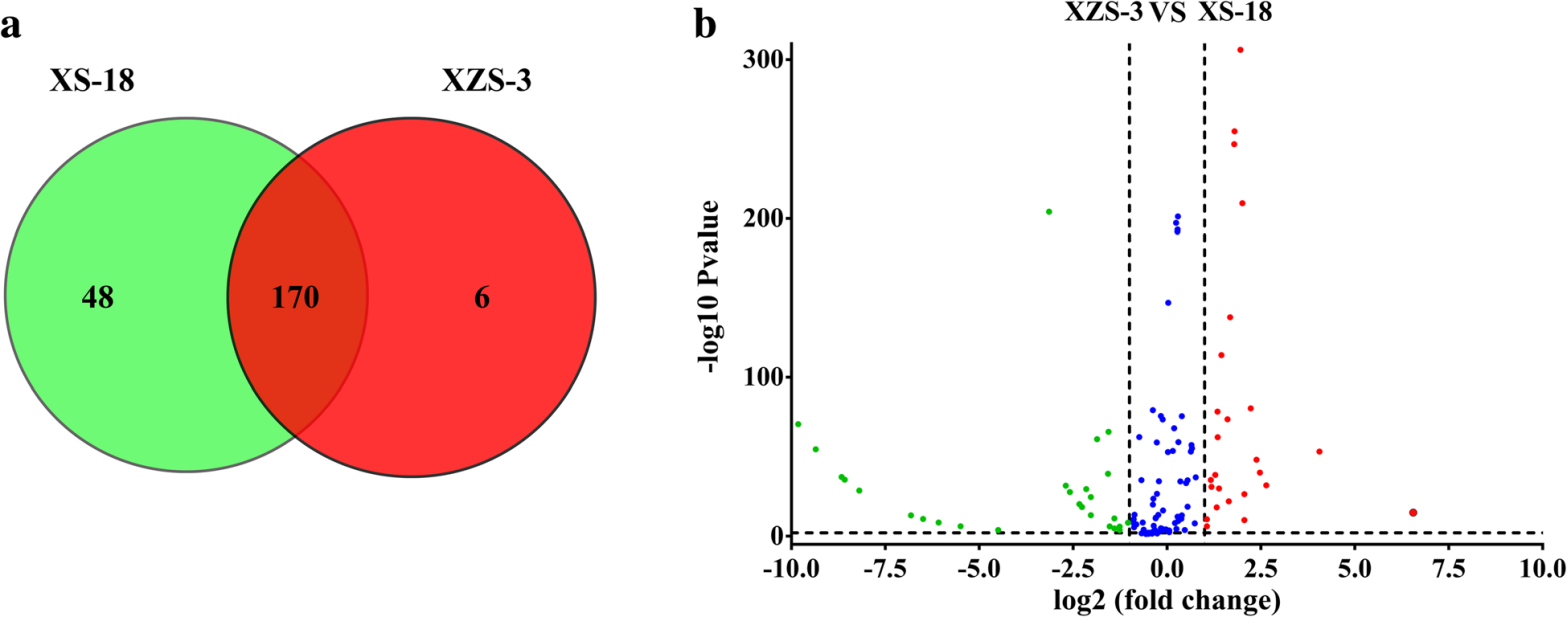
The differentially expressed miRNAs in XS-18 and XZS-3. **a** The Veen diagram of differentially expressed miRNAs. The number in each circle represented the expressed miRNAs in the corresponding sample, and in the overlapping part of the circles represented the co-expressed miRNAs between the XS-18 and XZS-3 samples. **b** The volcanic diagram of differentially expressed miRNAs. The X-axis represented fold change expression of miRNA in different libraries. The Y-axis represented significantly fold change expression of miRNA in statistics. The red dots and green dots represented significantly up-regulated and down-regulated miRNAs in XZS-3, respectively. The blue dots represented no significant difference

The normalized expression levels of miRNAs were compared between XS-18 and XZS-3 libraries to identify differentially expressed miRNAs. The known and novel miRNAs were followed to differential expression analysis criteria (qvalue < 0.01 and |log_2_ (fold change)| > 1). The Veen diagram analysis showed that 170 miRNAs co-expressed between the two libraries. Forty eight miRNAs (41 known and 7 novel miRNAs) presented specifically in XS-18 while 6 miRNAs (ib-miR162b, ib-miR164c, ib-miR166m, ib-miR166j, ib-miR414 and ib-miR845d) particularly expressed in XZS-3 (Fig. 5a; Additional file 10). Volcanic diagram showed a total of 121 of 224 (54.0%) miRNAs differentially expressed between the two libraries, among which 47 miRNAs were up-regulated and 74 miRNAs were down-regulated in XZS-3 compared to XS-18 (Fig. 5b; Additional file 10).

### Validation of ib-miRNAs and their target genes by qRT-PCR

**Fig. 6:**
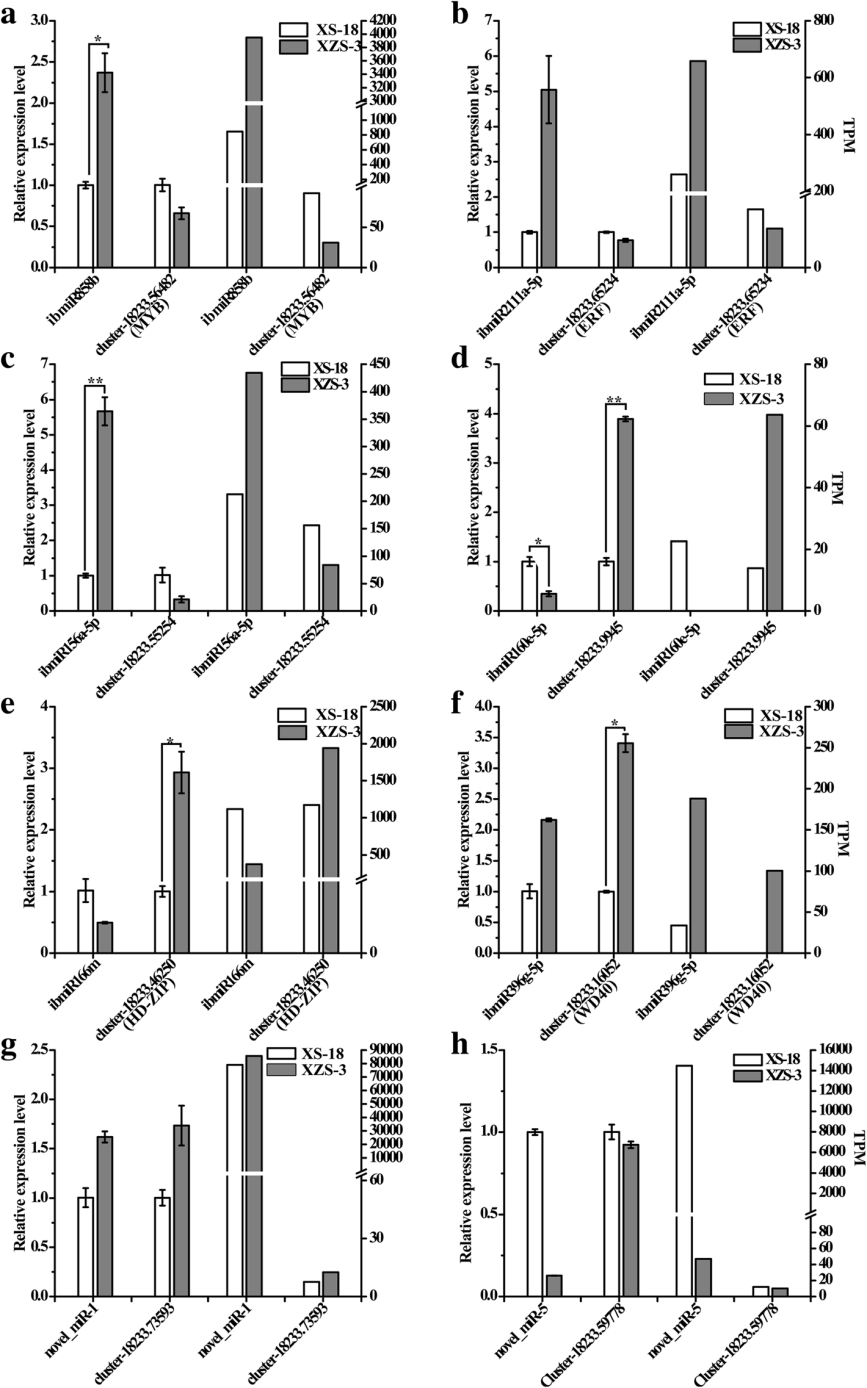
Comparison of the expression levels of the miRNAs and their corresponding target genes determined by qRT-PCR and deep sequencing. **a-h** The Y-axis on the left and right represented the relative expression levels determined by qRT-PCR and deep sequencing, respectively. *Ib5S* rRNA and *ibActin* were used as an internal control for miRNAs and targets, respectively. The expression levels of the miRNAs and targets in XS-18 were set as 1.0. Relative expression was calculated using the 2^-ΔΔCt^ method. The bars represented the mean ± SD values of three biological experiments. * and ** indicated a statistically significant difference between XS-18 and XZS-3 at *P* < 0.05 and 0.01 according to DMRT analysis, respectively

Eight differentially expressed miRNAs and their corresponding targets between XS-18 and XZS-3 were randomly selected for qRT-PCR to verify the results obtained from sRNA and degradome sequencing. In general, the expression patterns of the eight selected miRNAs showed by qRT-PCR were consistent with that showed by high-throughput sequencing, indicating that the sRNA sequencing data were reliable (Fig. 6). Up-regulated expression levels of ib-miR858b, ib-miR2111a-5p and ib-miR156a-5p were detected in XZS-3 compared to XS-18, whereas the abundance of their corresponding target genes *MYB*, *ERF* and *SPL* were down-regulated, respectively (Fig. 6a, b, c). The expression amounts of ib-miR160e-5p and ib-miR166m were decreased, while their corresponding target genes *ARF* and *HD-ZIP* had an increased expression in XZS-3 compared to XS-18 (Fig. 6d, e). The inversely correlated expression patterns between miRNA and their corresponding targets confirmed degradome sequencing data. However, the expression of ib-miR396g-5p, novel_miR-1 and novel_miR-5 showed the same trend with that of their corresponding transcripts, *Cluster-18,233.16052* (*WD40*), *Cluster-18,233.73593* (*MYB*) and *Cluster-18,233.59778* (*ARF*), respectively, suggesting that the transcripts may not be the cleaved targets of these miRNAs (Fig. 6f, g, h). The expressions of another five miRNAs listed in Table 1 were also verified by qRT-PCR (Additional file 11).

**Table 1:** Differentially expressed known miRNAs and their targets involved in anthocyanin biosynthesis in sweetpotato

	miRNA	Normalized expression		Target transcripts	Normalized expression		Transcripts Annotation	Degradome Detection
		XS-18	XZS-3		XS-18	XZS-3		
	ib-miR156a-5p	214.32	441.76	Cluster-18,233.55254	155.92	84.08		Yes
miR156	ib-miR156b	11.28	0	Cluster-18,233.20487	238.99	123.33	Squamosa promoter-binding-like protein; Transcription factor GAMYB	Yes
				Cluster-18,233.55255	112.25	80.82		Yes
	ib-miR156f-5p	11.28	0	Cluster-18,233.20485	239.94	219.41		Yes
				Cluster-18,233.20486	108.11	106.18		Yes
				Cluster-18,233.55250	133.91	189.22		
				Cluster-18,233.55252	10.51	46.1		Yes
				Cluster-18,233.55256	19.06	245.6		Yes
miR159	ib-miR159c-2	383.48	94.02	Cluster-18,233.73594	3.2	0	Transcription factor GAMYB	Yes
	ib-miR159c-3	225.57	47.01					No
	ib-miR159e	236.85	47.01	Cluster-18,233.73593	7.52	12.52		Yes
miR160	ib-miR160e-5p	22.56	0	Cluster-18,233.46832	698.25	1177.83	Auxin response factor 18	Yes
				Cluster-18,233.61692	0	0		Yes
				Cluster-18,233.71745	27.45	22		Yes
				Cluster-18,233.73056	14	3.79		Yes
				Cluster-18,233.9945	13.88	63.65		Yes
miR164	ib-miR164c	0	47.01	Cluster-18,233.42258	590.54	2040.1	NAC domain-containing protein; Homeobox-leucine zipper protein	Yes
				Cluster-18,233.42259	14.57	1332.45		Yes
				Cluster-23,975.1	20.31	6.74		Yes
	ib-miR164c-5p	676.72	329.07	Cluster-18,233.42257	1211.17	1972.55		Yes
miR165	ib-miR165a-3p.1	33.83	0	Cluster-18,233.49635	270.23	188.48	Homeobox-leucine zipper protein	Yes
	ib-miR165a-3p.2	33.83	0	Cluster-18,233.49636	605.46	559.74		Yes
miR166	ib-miR166i-2	33.84	0	Cluster-18,233.52940	207.95	246.36	Homeobox-leucine zipper protein	Yes
	ib-miR166i-3p	11.28	0	Cluster-18,233.49635	270.23	188.48		Yes
	ib-miR166m-2	1116.59	376.09	Cluster-18,233.46250	1171.11	1941.32		No
miR172	ib-miR172a-1	372.2	141.03	Cluster-18,233.34295	4.28	27.89	Ethylene-responsive transcription factor; Probable WRKY transcription factor	Yes
	ib-miR172b	11.28	0	Cluster-18,233.74211	151.94	101.57		Yes
	ib-miR172c	45.11	94.02	Cluster-18,233.74214	26.71	92.68		Yes
	ib-miR172c-3p			Cluster-18,233.40075	71.18	114.9		Yes
				Cluster-18,233.65234	80.65	53.88		Yes
	ib-miR172e-3p.2	146.62	329.07	Cluster-18,233.45961	39.2	48.52		Yes
		22.56	47.01	Cluster-18,233.48871	225.84	279.91		Yes
	ib-miR172i	135.34	329.07	Cluster-18,233.74217	0	66.97		Yes
miR319	ib-miR319a-2	6124.34	13,209.99					No
miR396	ib-miR396g-5p.2	33.84	188.04	Cluster-18,233.16052	0	100.39	WD repeat-containing protein	Yes
miR858	ib-miR858b	845.9	3948.9	Cluster-18,233.56482	25.72	7.37	Myb-related protein Myb4	Yes
	ib-miR858–2	11.28	47.01	Cluster-18,233.2285	180.33	29.79		Yes
miR2111	ib-miR2111-5p	3169.32	11,141.53	Cluster-18,233.65234	80.65	53.88	Ethylene-responsive transcription factor	Yes
	ib-miR2111a	169.18	517.12	Cluster-18,233.65234	80.65	53.88		Yes
	ib-miR2111a-5p	259.41	658.14	Cluster-18,233.65234	80.65	53.88		Yes

### GO and KEGG analysis of targets regulated by differentially expressed ib-miRNAs

**Fig. 7:**
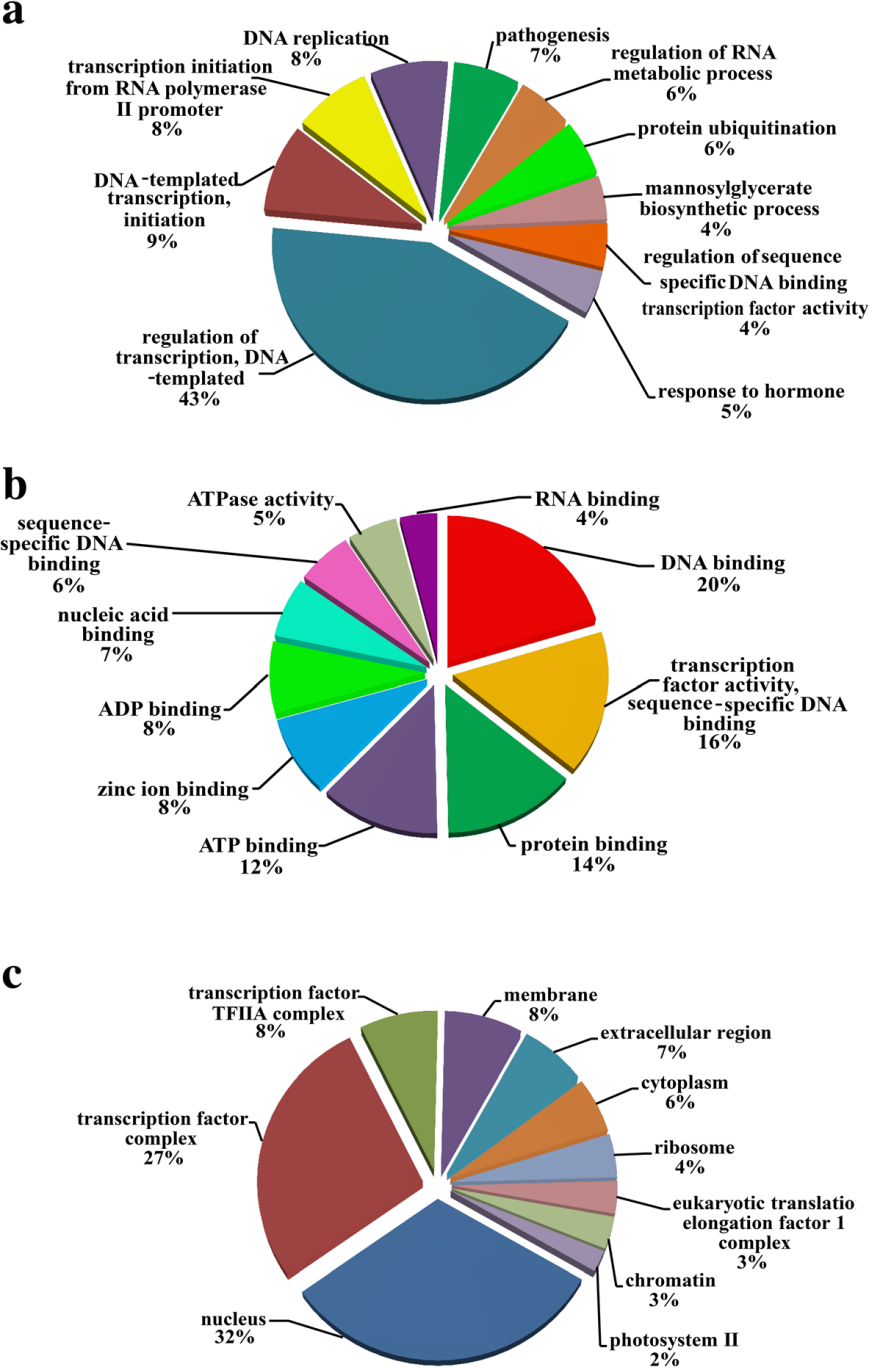
GO functional categorization of the target genes of the differentially expressed ib-miRNAs. The ten most dominant categories were performed according to biological process **a** molecular function **b** and cellular component **c**

A total of 3278 transcripts were predicted to be targets of the 224 miRNAs by computational program psRNATarget. Then, 180 targets identified by degradome sequencing were subjected to gene ontology (GO) analysis, and 124 of the 180 targets were found in GO database, which were involved in biological processes (79), molecular function (83), and cellular components (71) (Additional file 12). For biological processes category, more than 40% of the target genes were involved in regulation of transcription, DNA-templated annotations (GO:0006355), followed by DNA-templated transcription, initiation (GO:0006352) and DNA replication (GO:0006260) (Fig. 7a). In the molecular function category, the three most dominant terms were DNA binding (GO:0003677), sequence-specific DNA binding transcription factor activity (GO:0003700), and protein binding (GO:0005515) (Fig. 7b). Within the cell component category, the dominant two enrichments were the nucleus (GO:0005634) and the transcription factor complex (GO:0005667) (Fig. 7c).

**Fig. 8:**
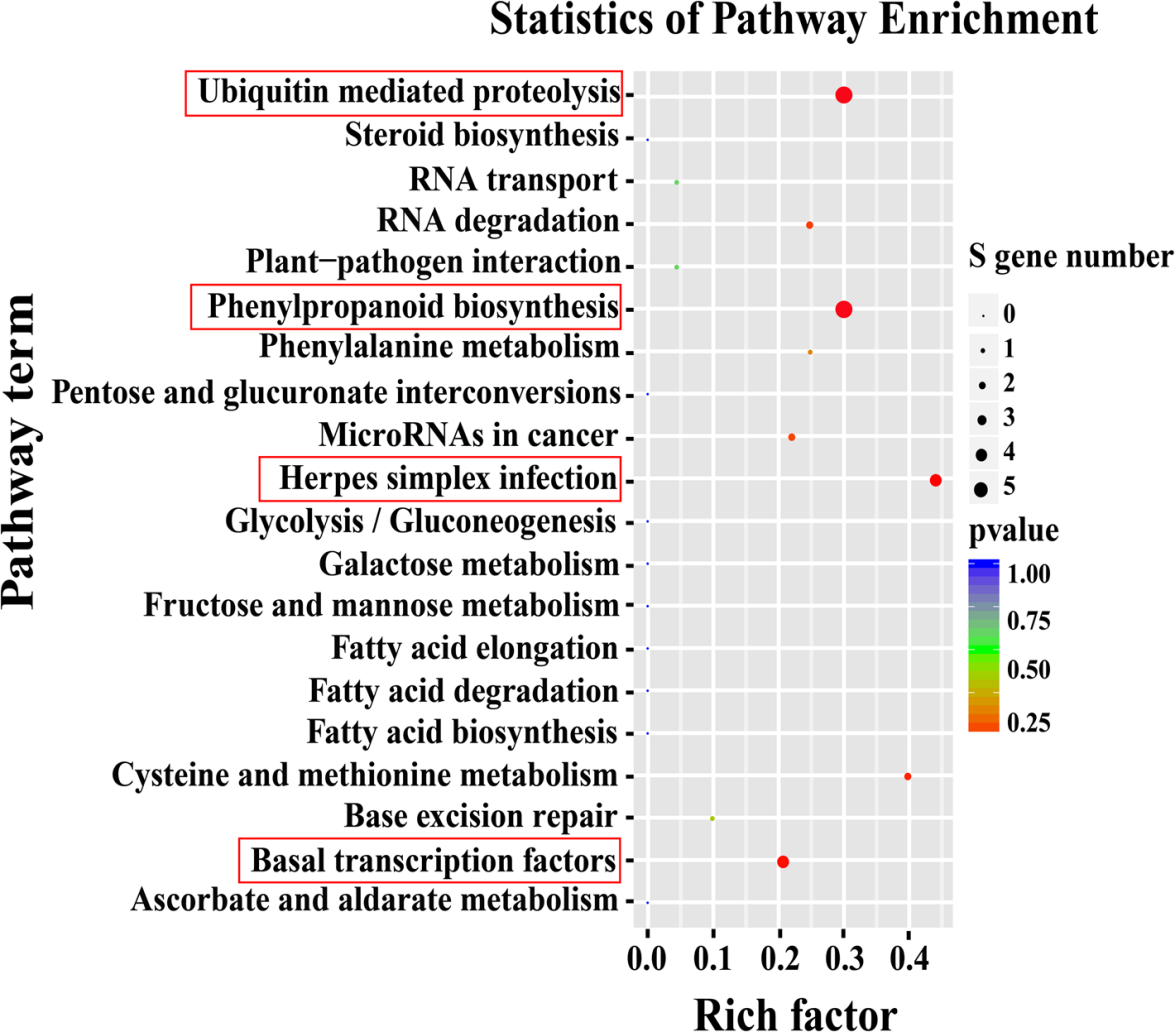
KEGG pathway analysis of the target genes regulated by the differentially expressed ib-miRNAs. The X-axis indicated the Rich factor, which referred to the ratio of the number of genes located in the differentially expressed gene to the total number of the annotated genes located in the pathway. The significance of the matched Rich factor was represented by the color of the *p*-values. The Y-axis indicated the names of enriched KEGG pathways. Each pathway was represented by a single circle and the circle size was proportional to the target gene numbers

**Fig. 9:**
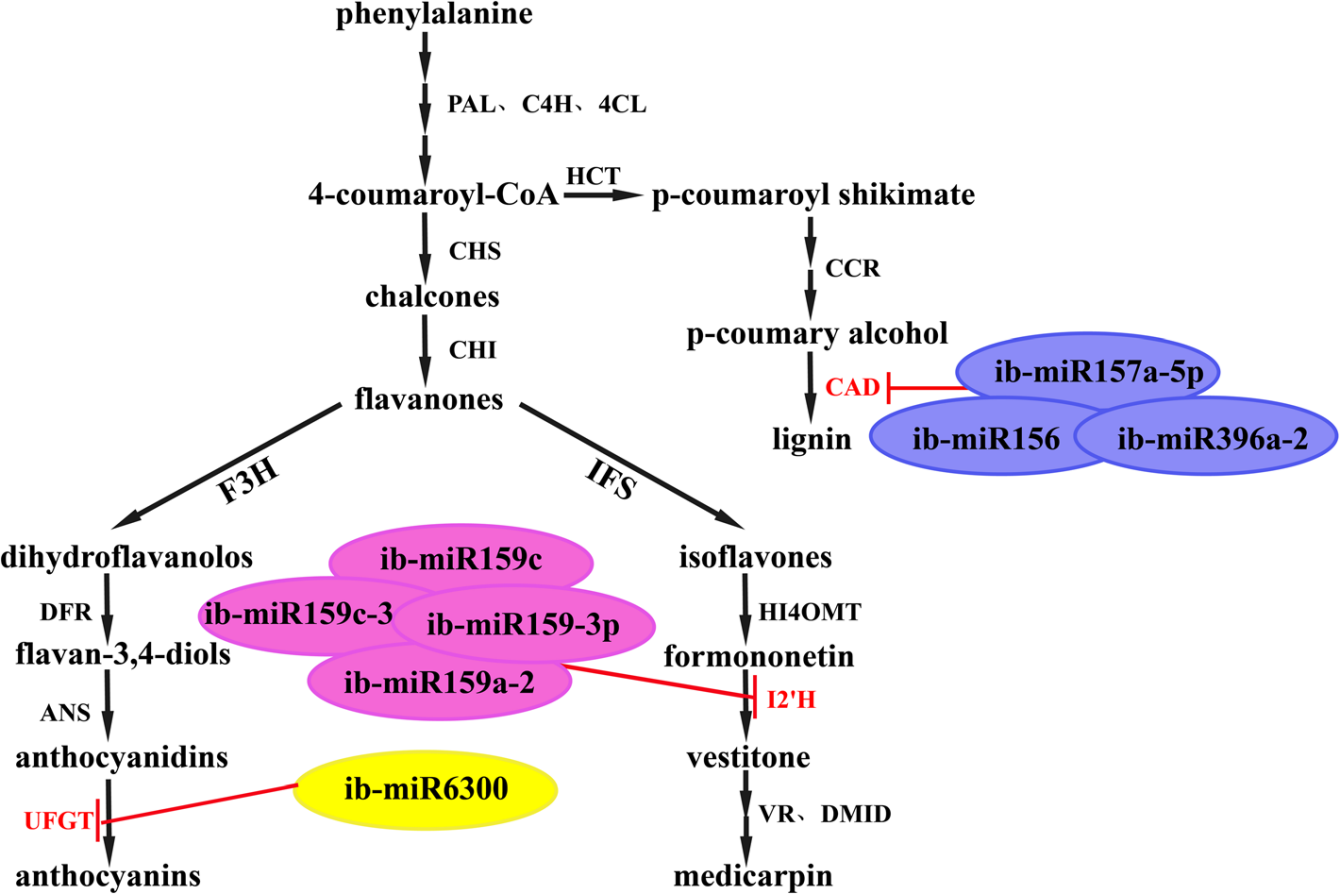
A putative model of ib-miRNAs and enzyme genes involved in phenylpropanoid pathway. MiRNA names in blue, pink and yellow circles represented miRNAs participated lignin, medicarpin and anthocyanin pathway, respectively. The potential target genes of ib-miRNAs were indicated in red

KEGG enrichment was carried out for pathway analysis. Twenty-two targets regulated by 12 different miRNAs were assigned to 11 KEGG pathways (Fig. 8; Additional file 13). Ubiquitin mediated proteolysis (ko04120, 5 targets) and phenylpropanoid biosynthesis (ko00940, 5 targets) were the most two enriched pathways, followed by herpes simplex infection (ko05168; 4 targets) and basal transcription factors (ko03022; 4 targets) (Fig. 8; Additional file 13). The anthocyanin biosynthesis originates from the general phenylpropanoid metabolic pathway [41]. In most species, genes and miRNAs associated with phenylpropanoid pathway may be involved in the anthocyanin biosynthesis. Based on the functions of the target genes in phenylpropanoid metabolism, a pathway panel for phenylpropanoid regulation was proposed in sweetpotato (Fig. 9). In particular, 8 ib-miRNAs (ib-miR157a-5p, ib-miR156, ib-miR396a-2, ib-miR159c; ib-miR159-3p, ib-miR159c-3, ib-miR6300, and ib-miR159a-2) participated in phenylpropanoid pathway in sweetpotato (Fig. 9), which may play significant roles in regulating the anthocyanin biosynthesis in sweetpotato.

### Identification of ib-miRNAs and their targets related to anthocyanin biosynthesis in sweetpotato

Identification of differentially expressed miRNAs in tuberous roots of WFSP and PFSP could help to better understand the biological function of miRNAs involved in anthocyanin biosynthesis. Combined with sRNA, degradome sequencing, GO and KEGG analysis, 26 of the 121 miRNAs differentially expressed miRNAs were identified to be potentially involved in anthocyanin biosynthesis in sweetpotato (Table 1, Additional file 14).

As expected, the previously reported miRNAs related to anthocyanin biosynthesis were also differentially expressed in this study (Table 1, Fig. 6). For instance, the expression levels of ib-miR156a-5p and ib-miR858b were higher in XZS-3 than XS-18. Aside from above mentioned miRNAs, the other 24 miRNAs representing 9 families were firstly identified to be putatively involved in anthocyanin biosynthesis (Table 1, Additional file 14). For example, the expression of ib-miR172c, ib-miR172e-3p.2, ib-miR172i, ib-miR172b, miR319, miR396 and miR2111 increased significantly in XZS-3. In particular, ib-miR164c was only expressed in XZS-3. However, family members of ib-miR159, ib-miR165, and ib-miR166 showed reduced or even no expression in XZS-3.

### Over-expression of ib-pri-MIR156 induced anthocyanin accumulation in *Arabidopsis*

**Fig. 10:**
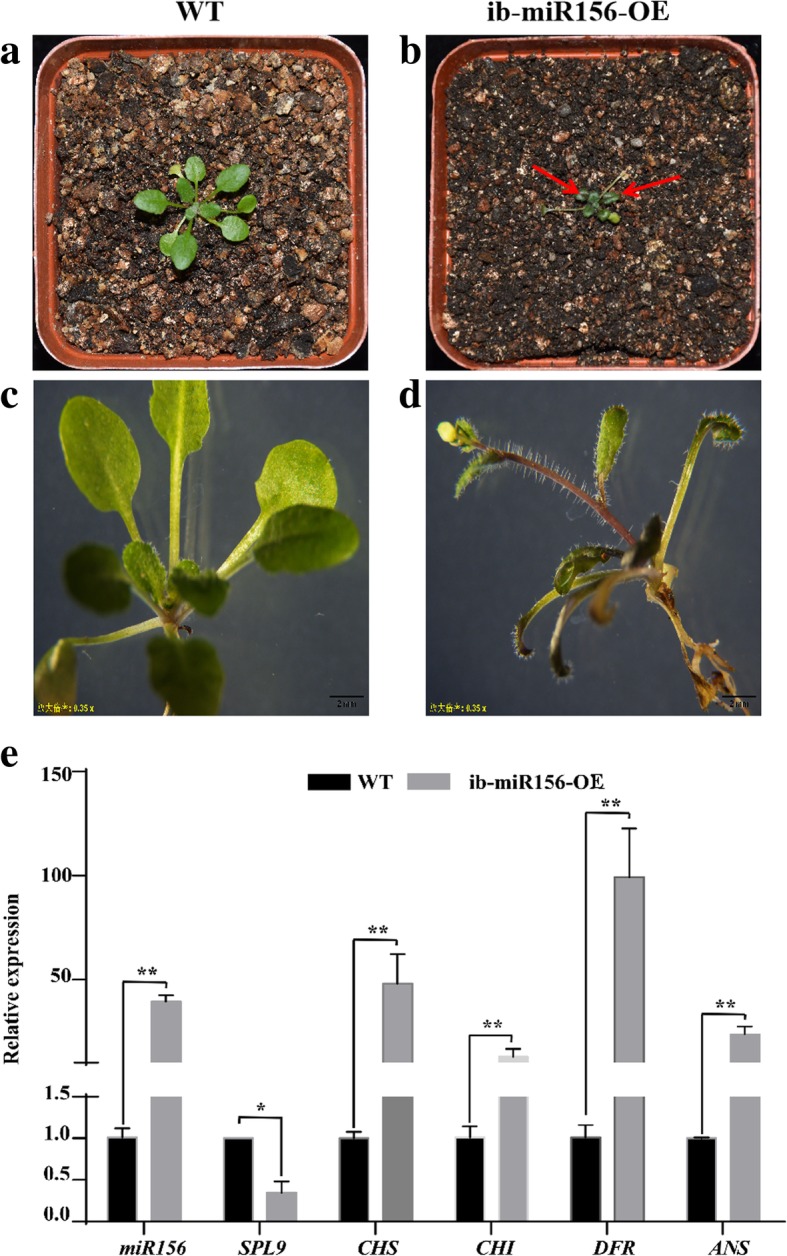
Phenotype and molecular analysis of ib-miR156 over-expressing transgenic *Arabidopsis*. **a, b** Phenotype of wild type *Arabidopsis* (WT) and ib-miR156-OE transgenic *Arabidopsis* plant in pots. **c, d** Phenotype of WT and ib-miR156-OE transgenic *Arabidopsis* plant under stereomicroscope (the magnification is 0.35 times). **e** the relative expression levels of ib-miR156, *SPL9*, *CHS*, *CHI*, *DFR* and *ANS* were measured by qRT-PCR. The expression level of each gene in WT was set as 1.0. Relative expression was calculated using the 2^-ΔΔCt^ method. The bars represented the mean ± SD values of three biological experiments. * and ** indicated a statistically significant difference between WT and ib-miR156-OE transgenic *Arabidopsis* at P < 0.05 and 0.01, respectively

The orthologous gene of miR156 in *Arabidopsis* has been demonstrated to regulate anthocyanin biosynthesis [14]. In this study, the inverse correlation between the accumulation of ib-miR156 and *ibSPL*, the targets of ib-miR156, in WFSP and PFSP suggested that ib-miR156 may also participate in anthocyanin biosynthesis. To test this hypothesis, the recombinant vector, pc2300-pOT2-ib-pri-MIR156, was generated and transformed into *Arabidopsis*. Interestingly, a purplish phenotype was observed in young seedlings of the ib-pri-MIR156 over-expressing *Arabidopsis* under normal growth conditions (Fig. 10). Analysis by qRT-PCR showed that the abundance of miR156 was greatly increased, whereas the expression of *SPL* decreased significantly in the transgenic *Arabidopsis* (Fig. 10e). Besides, upregulated expressions were detected for four structural genes related to anthocyanin biosynthesis, including *CHS*, *CHI*, *DFR* and *ANS* in the transgenic *Arabidopsis* (Fig. 10e). The results indicated that ib-pri-MIR156 positively regulated anthocyanin accumulation by repressing the expression of *SPL*.

## Discussion

### Identification of ib-miRNAs by high-throughput sequencing

MiRNAs, as the key post-transcriptional regulators, participate in various biological processes in plant. Recently, increasing evidences have showed that plant miRNAs are also involved in secondary metabolism [14, 38, 42]. Anthocyanin is one of the important secondary metabolite products. A number of miRNAs have been reported to play important roles in regulating anthocyanin biosynthesis in plant, such as miR156 [14], miR393 [43], miR828 [18, 25, 26], miR163 [44], miR165/166 [17] and miR858 [45]. However, the identification of miRNAs related to anthocyanin biosynthesis has not yet been reported in the tuberous root of sweetpotato.

In this study, a total of 191 known ib-miRNAs were identified from the WFSP and PFSP libraries by analysis of sRNA sequencing data. The number was much higher than previous reports on sweetpotato. In the first report on sweetpotato miRNAs, only 8 potential miRNA candidates were computationally identified [46]. Then, 24 known miRNAs were identified in Solexa Sequencing of Ningzishu 1 [36]. In the two studies, the number of miRNA identified is considerably limited because EST sequences were used as the references. The known miRNAs number of this study is comparable with the recent report on miRNAs responded to chilling stress of sweetpotato, in which 190 known miRNAs were found [47]. The research is based on Kazusa sweetpotato GARDEN database, which scaffold the genome sequences of a wild diploid ancestor of sweetpotato, *Ipomoea trifida*, although the total lengths of the assembled sequences was only 712 Mb and the size is far less than estimated genome sequence [48].

Most of the known miRNA families detected by Xie et al were also present in our dataset, except miR1508, miR5253, miR5298 and miR2911 [47]. On the other hand, we identified miR6478, miR6300, miR6173 and miR5508 which were not reported in sweetpotato previously. Since the expression levels of miRNAs existed temporal and spatial specificity, this result is not surprising [49]. Based on the hairpin structures of pre-miRNAs (Additional file 6), 33 novel candidate miRNAs were identified in this study. The number of novel miRNAs is clearly more than the research with the EST sequences as reference, but it is far less than the report of miRNAs responded to chilling stress of sweetpotato. Although the genomic sequence of Taizhong 6 sweetpotato cultivar was released last year, the incompleteness and inaccuracy of assembly limited its application as a good reference genome of hexaploid sweetpotato [50, 51]. In order to eliminate the difference caused by genetic background, species and development stages, the transcriptome sequences developed with the same sweetpotato cultivars with sRNA deep sequencing were used as the reference to predict the novel miRNAs in this study. Degradome sequencing has been successfully applied to identify miRNA targets in many plant species [52, 53]. In this study, a total 180 targets for 115 ib-miRNAs were obtained by the degradome sequencing approach. The number of targets supported by degradome was far less than that predicted by using computational psRNATarget. As expected, a majority target genes were transcription factors in plant species, and some ib-miRNA had more than one targets. Such as, ib-miR156a-5p and ib-miR5658 both had 10 targets and ib-miR159a had 6 targets. In addition, some ib-miRNAs belonging to the same family shared the same transcript. According to the annotation of targets, these target genes were found to participate in wide biological processes.

### Ib-miRNAs involved in anthocyanin biosynthesis in sweetpotato

Auxin is essential for plant development. High levels of auxin can repress the expression of the MBW complex and thereby regulate the biosynthesis of anthocyanin [54, 55]. *ARF, WRKY* and *HD-ZIP* genes play important roles in auxin-mediated signaling, which regulates anthocyanin biosynthesis through the Aux/IAA-ARF pathway in apple [56]. In plants, *ARF* genes were regulated by miR160 and miR390 [24, 57]. The WRKY mainly involved in auxin-mediated signaling and had considerable effect on flavonoid and anthocyanin biosynthesis [58, 59]. HD-ZIP TF plays critical roles in shoot apical meristem and organ polarity in plant [23]. Our previous study showed that blockage of miR165/166 caused the up-regulation of HD-ZIP TFs and increased IAA content accompanied by enhanced anthocyanin content in *Arabidopsis* [17]. In this study, the analysis of sRNA and degradome sequencing demonstrated that *WRKY* and *ARF* were targeted by ib-miR172e-3p and ib-miR160e-5p, respectively; while *HD-ZIPs* were targeted by ib-miR164c and ib-miR166m (Table 1). These results suggested that ib-miR172e-3p, ib-miR160e-5p, ib-miR164c and ib-miR166m might regulate anthocyanin biosynthesis through the auxin signaling.

*CAD* is one of the key enzyme genes of lignin biosynthesis [60], which has been found to be targeted by ib-miR156, ib-miR157 and ib-miR396 (Fig. 9). MiR156 and miR159 have been reported as potential regulators of secondary wall biosynthesis in *Acacia mangium* [61]. In this study, four members of ib-miR159 family were found to have a common novel target *I2’H* (Fig. 9), which can promote the accumulation of medicarpin [62]. MiR6300, only detected in few leguminous plants like *Catharanthus roseus* and *Camellia sinensis*, was found to target *UFGT* [63, 64]*.* Eight miRNAs were found to regulate the enzyme genes involved in the phenylpropanoid pathway in sweetpotato in this study. Based on the interactions of the miRNAs and enzyme genes in phenylpropanoid biosynthesis, we proposed a pathway panel for phenylpropanoid regulation in sweetpotato (Fig. 9).

The MYB TFs have been identified to be one of the major regulators in the pathway of anthocyanin biosynthesis [65–67]. For example, *AtPAP1* (*AtMYB75*) and its orthologs have been shown to effectively induce anthocyanin production in various plant species [68–70]. Over-expression of *MYB1* induced anthocyanin accumulation in sweetpotato and apple [65, 71]. *MYB* has been demonstrated to be regulated by many miRNA members. For instance, miR828 was involved in the anthocyanin production, trichome and cotton fiber development by regulating *MYB* in plant [16, 18, 72, 73]. MiR858 was found to play a positive role in the accumulation of anthocyanin by cleaving *MYBL2* in *Arabidopsis* [9]. MiR858 has also been identified to target up to 66 *MYB* members in apple [74, 75]. Similarly, our recent studies showed that Sl-miR858 regulated two *SlMYB* transcripts and functioned in anthocyanin accumulation in tomato [45]. In this study, *MYB* genes were also targeted by ib-miR858, ib-miR319, ib-miR159 and ib-miR156. Compared with WFSP, ib-miR858b was significantly up-regulated whereas its corresponding target gene *ibMYB* down-regulated in PFSP (Table 1; Fig. 6). The WD40 protein was reported to be essential and irreplaceable in the MYB-bHLH complex for anthocyanin biosynthesis in sweetpotato [76]. The transcript of *ibWD40* was not cleaved by ib-miR396g-5p in degradome sequencing, although it was a potential target of ib-miR396g-5p predicted by psRNATarget (Additional file 7). Ib-miR396g-5p and *ibWD40* both expressed higher in PFSP than in WFSP showed by qRT-PCR (Fig. 6). One possible reason is that the miRNAs may impose a translational regulation on their targets, which could not be detectable by degradome sequencing. Transcription factor bHLH is essential for the activity of the R2R3-MYB partner and can directly bind to the promoters of *DFR* and *UFGT* to activate or inhibit anthocyanin biosynthesis [77, 78]. However, none of miRNA was identified to target *bHLH* in this study. The enzyme gene *LcUFGT* plays a major role in anthocyanin accumulation [79]. In sweetpotato, we found ib-miR6300 directly targeted *ibUFGT*, suggesting that b-miR6300 may participate in anthocyanin biosynthesis by regulating *ibUFGT* (Additional file 9). However, the regulatory mechanism needs further validation.

SPLs are transcription factors widely existing in plants, which play an important role in plant growth and development, primary and secondary metabolism, as well as other biological processes. In *Arabidopsis*, *SPL* was demonstrated to be regulated by miR156, and acted as negative regulators of anthocyanin accumulation by destabilization of the MBW complex in *Arabidopsis* [14]. In this study, ib-miR156a-5p was identified to cleave *ibSPL* genes (Additional file 9). The results of sRNA sequencing and qRT-PCR showed that miR156a-5p significantly up-regulated whereas its target *ibSPL* expressed down-regulated in PFSP compared with WFSP (Fig. 6). By over-expressing ib-miR156 in *Arabidopsis*, a purple phenotype with high anthocyanin accumulation in the main stem of transgenic *Arabidopsis* was observed. The results of qRT-PCR showed that the over-expression of ib-miR156 in *Arabidopsis* strongly suppressed the abundance of *SPL* (Fig. 10), which consistent with the results of Gou et al. [14]. In addition, up-regulated transcripts for four structural genes of anthocyanin pathway were detected in ib-miR156 over-expressing transgenic plant (Fig. 10), suggesting that ib-miR156 may modulate anthocyanin biosynthesis through regulation of the structure genes in the phenylprepanoid pathway. These results indicated that the regulatory function of ib-miR156 is similar to that of at-miR156.

### Putative ib-miRNA-target model involved in anthocyanin biosynthesis in sweetpotato

**Fig. 11:**
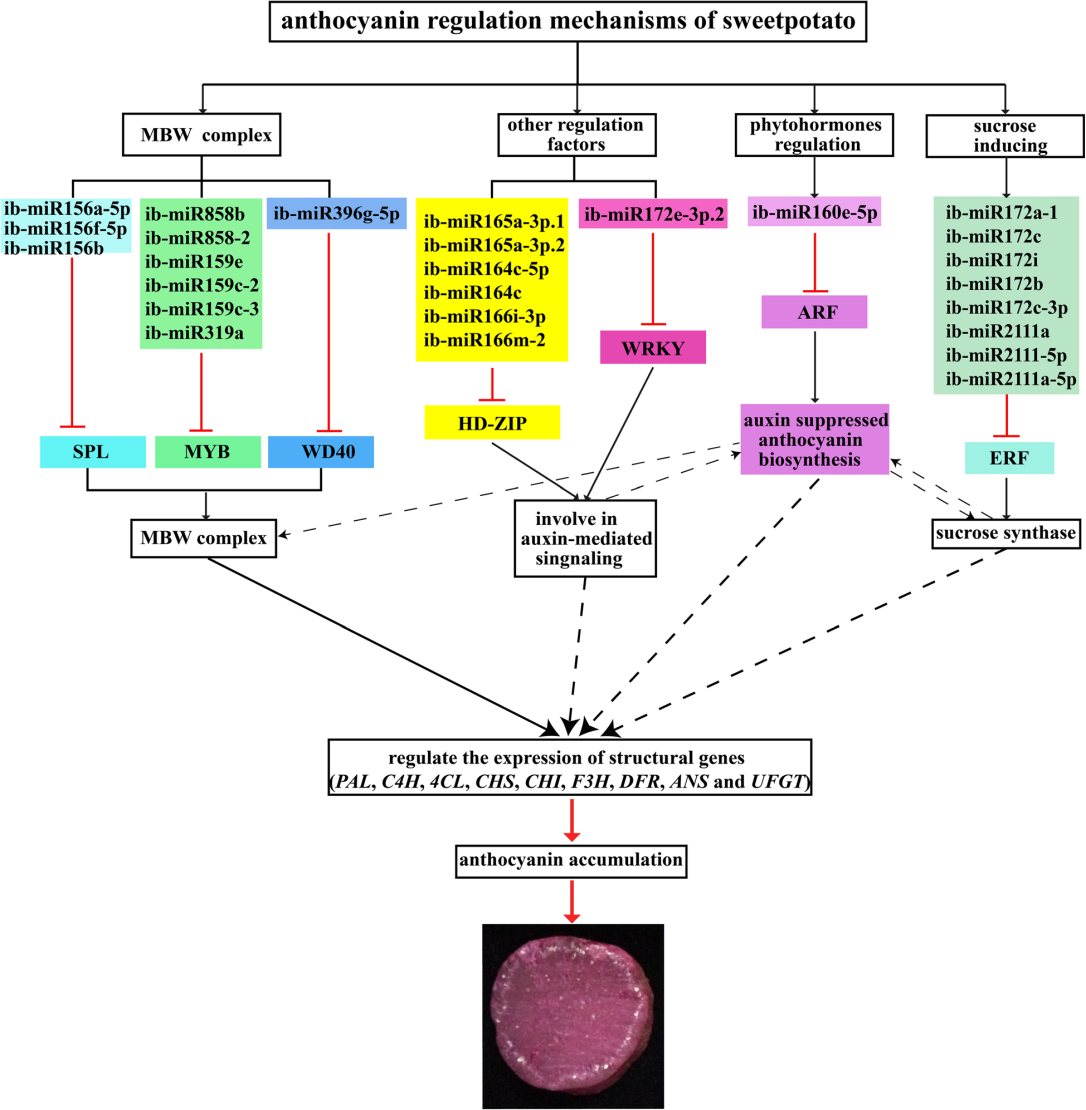
A proposed interaction model between miRNAs and their corresponding target genes associated with the genes in sweetpotato anthocyanin biosynthesis. The solid and dashed arrowhead lines represented the proved and potential regulatory roles, respectively

Anthocyanin biosynthesis was modulated by regulating genes, including *MYB*, *WDR*, *bHLH*, *SPL*, *ARF* and *MADS-box*. In addition, sucrose synthase, ABC transporter and sugar/inositol transporter were also potentially participated in anthocyanin biosynthesis. In this study, according to the annotation of targets, we found that 26 differentially expressed miRNAs and 36 their corresponding regulator genes were more likely to participate in anthocyanin biosynthesis in sweetpotato (Table 1, Additional file 14). Based on the present data and previous reports, a possible ib-miRNA-target model related to anthocyanin biosynthesis was proposed. As shown in Fig. 11, miRNA families of ib-miR156, ib-miR159, ib-miR396 and ib-miR858 potentially targeted *SPL*, *MYB* and *WDR* to shape a regulatory MBW complex; miRNA families of ib-miR164, ib-miR165/166 and ib-miR172e-3p.2 possibly targeted *WRKY* and *HD-ZIP* to involve in auxin signaling; ib-miR160, miRNA families of ib-miR172 and ib-miR2111 might regulate *ARF* and *ERF* to activate auxin-mediated signaling and sucrose signaling. All of these biological processes would influence the expression of structural genes, and subsequently form a complex regulatory network to modulate anthocyanin biosynthesis.

## Conclusions

In summary, this is the first report on systematic identification, expression analysis and potential roles of miRNAs and their targets in regulating anthocyanin biosynthesis in tuberous roots of sweetpotato. A total of 26 differentially expressed miRNAs and 36 corresponding targets were more likely to be related to anthocyanin biosynthesis by bioinformatic analysis and expression validation. By *Agrobacterium* mediated genetic transformation, ib-miR156 over-expressing transgenic *Arabidopsis* with purplish phenotype were obtained. The expression amount of ib-miR156 was up-regulated in the transgenic lines, while its target *SPL* showed down-regulated expression. Four anthocyanin-specific enzyme genes, *CHS*, *CHI*, *DFR* and *ANS*, expressed significantly higher in transgenic *Arabidopsis* than in the wild type plants (WT), suggesting that ib-miR156 could positively mediate anthocyanin biosynthesis by modulating related structural genes. Base on above results, a putative ib-miRNA-target model associated with anthocyanin biosynthesis in sweetpotato was proposed. Our findings provided comprehensive information for anthocyanin-specific miRNAs and their targets, as well as a starting point for mechanism investigation of miRNAs in anthocyanin biosynthesis in sweetpotato.

## Materials and methods

### Plant materials

Sweetpotato (*Ipomoea batatas* L.) cultivars of white-fleshed Xushu-18 (XS-18) and purple-fleshed Xuzishu-3 (XZS-3) were used for sRNA and degradome sequencing. The cultivars were provided by the Sweetpotato Research Institute in Jiangsu province of China and grown in the experimental field of the Shanxi Agricultural University (Taigu, China) under the normal conditions. After root formation at the stage of 90 days, white and purple tuberous roots with similar size and shape were selected. After gently washed, the roots were immediately frozen by liquid nitrogen and stored at − 80 °C freezer. We mixed three tuberous roots samples together as one biological replicate to eliminate effects of individual genetic variance, respectively. In total, three independent biological replicates were collected for each sample.

### Small RNA library construction and sequencing

Total RNAs were extracted from sweetpotato roots using Trizol reagent (Invitrogen, USA) according to the manufacturer’s instructions. Total RNA from each genotype was isolated in three biological replicates and the quality and quantity of RNAs were measured with the NanoPhotometer® spectrophotometer (IMPLEN, CA, USA). Then, two sequencing libraries (XS-18 and XZS-3) were constructed using NEBNext® Multiplex Small RNA library Prep Set for Illumina® (NEB, USA.) following the manufacturer’s recommendations and sequenced on an Illumina Hiseq 2000 platform at Novogene Bioinformatics Institute, Beijing, China.

### Identification and expression analysis of sweetpotato miRNAs using deep sequencing

The process for known and novel miRNAs in sweetpotato tuberous roots was performed following the previous reported approach [39]. Firstly, clean reads were obtained from raw data by removing reads containing ploy-N, larger with 5’adapter contaminants, without 3’adapter or the insert tag, containing ploy A or T or G or C and low quality reads. Sequences smaller than 18 nucleotide (nt) and larger than 30 nt were also removed. Then the sRNA tags were mapped to reference sequence by Bowtie [80] without mismatches allowed. The transcriptome sequences using the same sweetpotato cultivars (XS-18 and XZS-3) with sRNA deep sequencing were developed in our lab (not published). The assembled sequences from the transcriptome database were used as reference sequence for predicting miRNA precursors. The mapped sequences were subjected to BLASTn search against Repeat Masker (http://www.repeatmasker.org/) and Rfam database (http://rfam.xfam.org/) to remove tags originating from protein-coding genes, repeat sequences, rRNA, tRNA, snRNA, and snoRNA, or those types of data from the specified species itself. Usually, we followed the following priority rule: known miRNA > rRNA > tRNA > snRNA > snoRNA > repeat > gene > NAT-siRNA > gene > novel miRNA > ta-siRNA. The remaining reads were aligned with the miRNA sequences deposited in the miRBase 22.1 database (http://www.mirbase.org/) [81]. The matched sequences with no mismatches were considered to be known miRNAs. The unaligned reads were then subjected to software miREvo and miRdeep2 to predict novel miRNA candidates through exploring the secondary structure, the Dicer cleavage site and the minimum free energy [82]. The stem loop hairpin structures of pre-miRNAs were also predicted using RNAfold software. MiRNA expression levels were estimated by TPM (transcript per million) through the following Normalization formula: Normalized expression = mapped read count/total reads*1000000 [83].

To reveal the differentially expressed miRNAs related to anthocyanin biosynthesis between XS-18 and XZS-3, the miRNAs expression was analyzed using the DEGseq R package, respectively. *P*-value was adjusted using qvalue [84]. Qvalue < 0.01 and |log2 (fold change)| > 1 was set as the threshold for significantly differential expression by default.

### Degradome library construction, sequencing and data analysis

To identify potential target mRNAs for sweetpotato miRNAs, a degramdome library was constructed using the mixed roots of XS-18 and XZS-3. Firstly, by using the Oligotex kit (Qiagen, Germany), 200 μg of total RNA was used for extracting poly (A) RNA, which was ligated to a 5′ adapter with an EcoP15 I recognition site in its 3′ end. After ligated, the first-strand cDNA was generated and amplify by PCR. The PCR product was purified and digested with PAGE-gel and EcoP15 I, respectively. Then, the EcoP15 I cleaved fragments were ligated to a 3′ double-strand DNA adapter and followed by PAGE-gel purification to obtain the ligated products. After PCR amplification, PAGE-gel was used for the third time to purify the final products. Finally, the purified cDNA library was ready for deep sequencing on Illumina HiSeq2000 sequencing system (LC-BIO Sciences, China).

After sequencing, the adapter sequences, low-quality reads and N-containing fragments were filtered from the raw reads. The remaining sequences mapped to the sweetpotato transcriptome database of XS-18 and XZS-3 were used to identify potentially cleaved targets by CleaveLand4 pipeline as previously described [85]. The degradome event mediated by sRNA was categorized as 0, 1, 2, 3, and 4 based on the abundance of its cleaved tag reads across the target transcript [86]. Category 0: Abundance equal to the maximum of the target transcript abundance and the cleaved tag reads had only one maximum value. Category 1: Abundance equal to the maximum of the target transcript abundance, and the cleaved tag reads had more than one maximum value. Category 2 represented the cleaved tag abundance was less than the maximum but higher than the mean of the transcript abundance. Category 3: the cleaved tag abundance was less than or equal to the mean of the transcript abundance. Category 4 represented the depth of the position equal to 1. T-plots were built to analyze the miRNA targets and RNA degradation patterns according to the distribution and abundances along these transcripts. The potential targets of miRNAs were analyzed by PAREsnip software with P-value < 0.05 [87].

### GO enrichment and KEGG pathway analysis

GO terms of the miRNAs targets were annotated according to their biological role, molecular function and cellular component by using the online GO analysis tool (http://www.geneontology.org/page/go-enrichment-analysis) [88]. The statistical enrichment of the candidate target genes in KEGG pathways was set by KOBAS software [89, 90].

### Construction of ib-pri-MIR156 over-expressing vector and genetic transformation of *Arabidopsis*

Ib-pri-MIR156 was isolated from PFSP, and inserted into a pOT2 vector between the 35S2 promoter and the 35S terminator. Then, the pOT2 was mobilized into a modified pCAMBIA2300 binary vector to construct over-expressing vector pc2300-pOT2-ib-pri-MIR156. The vector was introduced into *Agrobacterium* GV3101 and followed by *Agrobacterium* mediated transformation to obtain transgenic *Arabidopsis* [91]. All the primers used for plasmid construction were listed in Additional file 15.

### Analysis of the expressions of ib-miRNAs, corresponding targets and anthocyanin-specific enzyme genes by qRT-PCR

The expressions of miRNAs and their corresponding targets were evaluated by qRT-PCR. Total RNA was extracted from XS-18 and XZS-3 by using Trizol (Invitrogen, USA). For miRNA expression analysis, the specific stem loop RT, forward and universal reverse primers were designed according to the reported method with some modifications [92]. One μg of DNase treated total RNA was used for cDNA synthesis following the procedures of PrimeScript™ 1st Strand cDNA synthesis kit (TaKaRa, China). All qRT-PCR analysis was carried out using SYBR *Premix Ex Taq*™ (Tli RNaseH Plus) (TaKaRa, China) and performed on a Bio-Rad CFX96 Real-Time PCR Systems (Bio-Rad, USA). For sweetpotato, the *ib5S* and *ibActin* gene were used as internal reference for accurate normalization in each reaction for the miRNA, and target genes, respectively [93]. For transgenic *Arabidopsis* over-expressing miR156, the *atU6* and *atActin* were used. The relative expression levels were calculated using the 2^-ΔΔCt^ method [94]. Each sample from three biological replicates was performed in triplicate. Results were presented as means ± SD. The method of Dunnett’s two-tailed t-test was used to conduct the statistical analysis of RT-PCR results, and the statistical significant differences were shown at *p* ≤ 0.05 (marked *) and *p* ≤ 0.01 (marked **). The sequences for all the primers were listed in Additional file 15.

## Additional files

Additional file 1: Statistics of sRNA sequences and transcriptome mapping of the libraries in sweetpotato. (DOC 32 kb)

Additional file 2: Distribution of unique sRNA sequences in different categories in sweetpotato. (DOC 32 kb)

Additional file 3: Detailed information of the known miRNAs identified in sweetpotato. (XLS 69 kb)

Additional file 4: Normalized read counts of the known miRNAs. (XLS 37 kb)

Additional file 5: Detailed information of the novel miRNAs identified in sweetpotato. (XLS 36 kb)

Additional file 6: Secondary structures of novel miRNA precursors. (TIF 269 kb)

Additional file 7: Predicted target genes of miRNAs identified in sweetpotato by psRNATarget. (XLS 935 kb)

Additional file 8: Statistics of degedrom sequencing reads in sweetpotato. (DOC 22 kb)

Additional file 9: Target genes of miRNAs identified in sweetpotato using degradome sequencing. (XLS 126 kb)

Additional file 10: List of differentially expressed miRNAs between XS-18 and XZS-3. (XLS 33 kb)

Additional file 11: Comparison of the expression levels of the miRNAs determined by qRT-PCR and deep sequencing. (TIF 901 kb)

Additional file 12: GO analysis for the targets of differentially miRNAs identified by degradome sequencing in sweetpotato. (XLS 63 kb)

Additional file 13: KEGG analysis for the targets of differentially miRNAs identified by degradome sequencing in sweetpotato. (XLS 24 kb)

Additional file 14: The miRNAs and corresponding target genes potentially related to anthocyanin biosynthesis in sweetpotato. (XLS 24 kb)

Additional file 15: List of primers used in this study. (XLS 26 kb)

## Abbreviations

ANS: Anthocyanidin synthase
ARF: Auxin response factor
CAD: Cinnamyl alcohol dehydrogenase
CHI: Chalcone isomerase
CHS: Chalcone synthase
DFR: Dihydroflavonol 4-reductase
ERF: Ethylene-responsive transcription factor
EST: Expressed sequence tag
F3’5’H: Flavonoid 3′,5′-hydroxylase
F3’H: Flavanone 3-hydroxylase
F3H: Flavanone 3-hydroxylase
GO: Gene ontology
HD-ZIP: Homeodomain-leucine zipper protein
I2’H: Isoflavone 2′-hydroxylase
IAA: Indole-3-acetic acid
KEGG: Kyoto encyclopedia of genes and genomes
PAL: Phenylalanine ammonia lyase
SPL: Squamosa promoter-binding protein-like
sRNA: small RNA
TF: Transcript factor
UFGT: UDP-glycose flavonoid glycosyltransferase
WDR: WD repeat-containing proteins
