# Supplementary material for: Uncovering anthocyanin biosynthesis related microRNAs and their target genes by small RNA and degradome sequencing in tuberous roots of sweetpotato
Source: BMC Plant Biol. 2019 Jun 3;19:232. doi: 10.1186/s12870-019-1790-2 (PMC6547535; doi:10.1186/s12870-019-1790-2)
Supplement: Supplementary file 1 — Statistics of sRNA sequences and transcriptome mapping of the libraries in sweetpotato. (DOC 32 kb) [file 12870_2019_1790_MOESM1_ESM.doc]

**Additional file 1: Statistics of sRNA sequences and transcriptome mapping of the libraries in sweetpotato**.

| **Library** | **RRN03889** | | **RRN03888** | |
| --- | --- | --- | --- | --- |
| Sample | XZS-3 | XZS-3 (percent) | XS-18 | XS-18 (percent) |
| total_reads | 28,947,914 | 100.00% | 27,705,914 | 100.00% |
| N% > 10% | 3,339 | 0.01% | 697 | 0.00% |
| low quality | 126,065 | 0.44% | 100,140 | 0.36% |
| 5_adapter_contamine | 32,017 | 0.11% | 35,905 | 0.13% |
| 3_adapter_null or insert_null | 1,446,417 | 5.00% | 729,538 | 2.63% |
| with ployA/T/G/C | 8,369 | 0.03% | 14,000 | 0.05% |
| clean reads | 27,331,707 | 94.42% | 26,825,634 | 96.82% |
| Total sRNA | 17,207,939 | 62.96% | 18,963,838 | 70.69% |
| Mapped sRNA | 15,764,489 | 91.61% | 13,904,486 | 73.32% |
