# Supplementary material for: Uncovering anthocyanin biosynthesis related microRNAs and their target genes by small RNA and degradome sequencing in tuberous roots of sweetpotato
Source: BMC Plant Biol. 2019 Jun 3;19:232. doi: 10.1186/s12870-019-1790-2 (PMC6547535; doi:10.1186/s12870-019-1790-2)
Supplement: Supplementary file 2 — Distribution of unique sRNA sequences in different categories in sweetpotato. (DOC 32 kb) [file 12870_2019_1790_MOESM2_ESM.doc]

**Additional file 2: Distribution of unique sRNAs sequences in different categories in sweetpotato**.

| **Types** | **XZS-3** | **XZS-3 (percent)** | **XS-18** | **XS-18 (percent)** |
| --- | --- | --- | --- | --- |
| total | 15,764,489 | 100% | 13,904,486 | 100% |
| known_miRNA | 15,500 | 0.10% | 52,540 | 0.38% |
| rRNA | 2,729,754 | 17.32% | 2,111,142 | 15.18% |
| tRNA | 2 | 0.00% | 0 | 0.00% |
| snRNA | 1,604 | 0.01% | 1,540 | 0.01% |
| snoRNA | 10,394 | 0.07% | 7,648 | 0.06% |
| novel_miRNA | 4,793 | 0.03% | 27,962 | 0.20% |
| TAS | 24 | 0.00% | 121 | 0.00% |
| other | 13,002,418 | 82.48% | 11,703,533 | 84.17% |
