# Supplementary figures and images for: Uncovering anthocyanin biosynthesis related microRNAs and their target genes by small RNA and degradome sequencing in tuberous roots of sweetpotato
Source: BMC Plant Biol. 2019 Jun 3;19:232. doi: 10.1186/s12870-019-1790-2 (PMC6547535; doi:10.1186/s12870-019-1790-2)

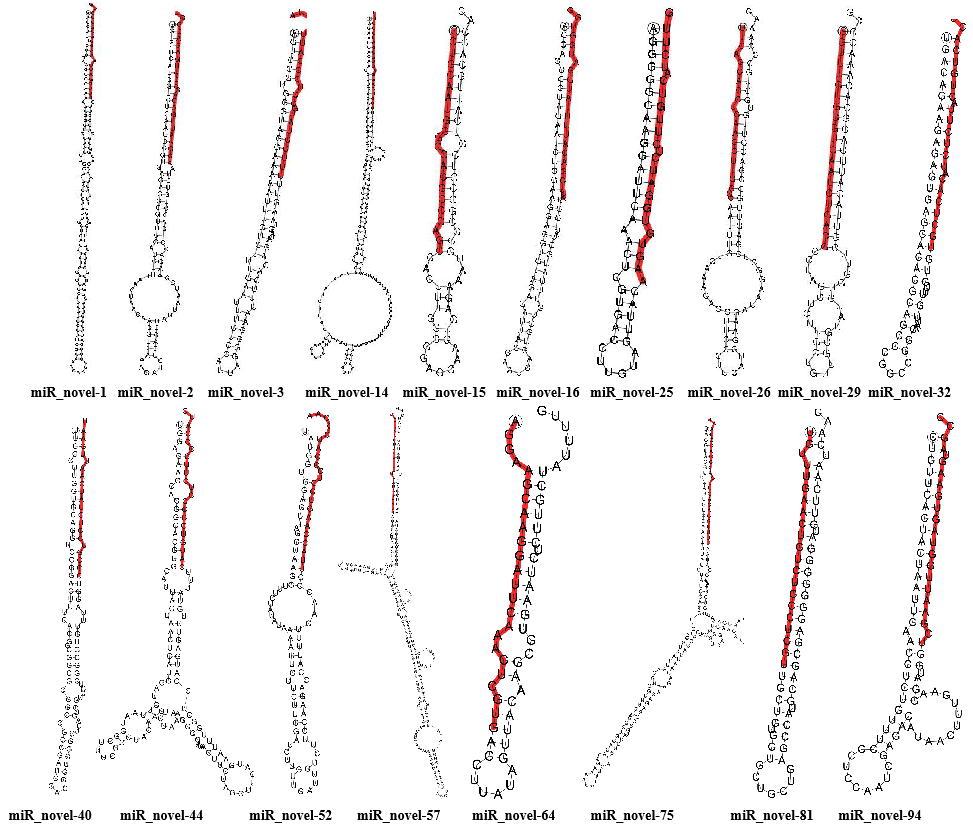

Supplement: Supplementary file 6 — Secondary structures of novel miRNA precursors. (TIF 269 kb) [file 12870_2019_1790_MOESM6_ESM.tif]

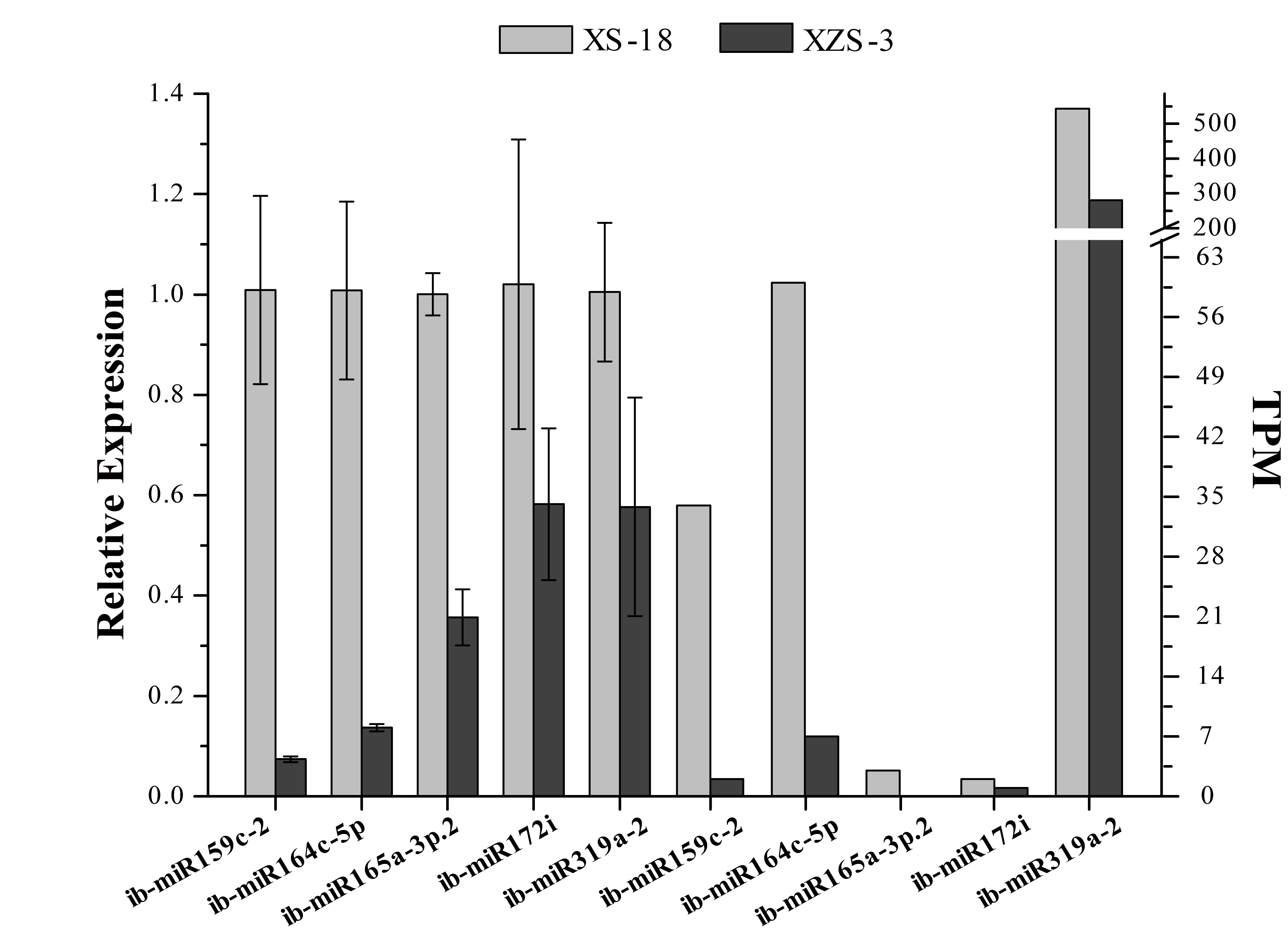

Supplement: Supplementary file 11 — Comparison of the expression levels of the miRNAs determined by qRT-PCR and deep sequencing. (TIF 901 kb) [file 12870_2019_1790_MOESM11_ESM.tif]
