# Supplementary material for: Uncovering anthocyanin biosynthesis related microRNAs and their target genes by small RNA and degradome sequencing in tuberous roots of sweetpotato
Source: BMC Plant Biol. 2019 Jun 3;19:232. doi: 10.1186/s12870-019-1790-2 (PMC6547535; doi:10.1186/s12870-019-1790-2)
Supplement: Supplementary file 8 — Statistics of degedrom sequencing reads in sweetpotato. (DOC 22 kb) [file 12870_2019_1790_MOESM8_ESM.doc]

**Additional file 8: Statistics of degedrom sequencing reads in sweetpotato.**

| **Sample** | **tuberous root (number)** | **tuberous root (ratio)** |
| --- | --- | --- |
| Raw Reads | 14,514,990 | / |
| reads < 15nt after removing 3 adaptor | 54,975 | 0.38% |
| Mappable Reads | 14,460,015 | 99.62% |
| Unique Raw Reads | 1,904,303 | / |
| Unique reads < 15nt after removing 3 adaptor | 18,111 | 0.95% |
| Unique Mappable Reads | 1,886,192 | 99.05% |
| Transcript Mapped Reads | 9,001,937 | 62.02% |
| Unique Transcript Mapped Reads | 766,328 | 40.24% |
| Number of input Transcript | 111,017 | / |
| Number of Coverd Transcript | 53,700 | 48.37% |
